# The Following List of Deaths Is Given by the Bills of Mortality, for the Last Three Months

**Published:** 1799-06

**Authors:** 


*The following List of Deaths is given by the Bills of Mortality, for the last three Months,*

| | | | |
|---|---|---|---|
| Abfcefs | 5 | Jaundice | 21 |
| Abortive | 10 | Inflammation | 194 |
| Aged | 439 | Liver-grown | 4 |
| Apoplexy | 32 | Lunatic | 32 |
| Afthma | 295 | Meafles | 24 |
| Bleeding | 1 | Mifcarriage | 1 |
| Brain Fever | 1 | Mortification | 43 |
| Cancer | 13 | Palfy | 19 |
| Child-bed | 46 | Pleurify | 8 |
| Confumption | 1353 | Rupture | 3 |
| Convulfions | 1033 | Small-Pox | 410 |
| Croup | 4 | Still-born | 161 |
| Dropfy | 220 | Stone | 1 |
| Fever | 510 | Suddenly | 42 |
| Gout | 25 | Teeth | 108 |
| Gravel | 2 | Thrufh | 6 |
| Hooping-Cough | 190 | Water in the Head | 15 |

According to this ftatement, if we could rely upon its accuracy, the total number of deaths in London, during the three fpring-months, amounted to 5271. Among thefe, no lefs than 1353, or upwards of one fourth part, died of confumption!—Suppofing the population of the country to confift of ten millions of inhabitants, the thirtieth part of whom in the order of things, die annually, it would follow, that this exterminating difeafe cuts off more than 80,000 perfons every year, in Great Britain alone; and thefe generally in the prime of life, when the community ought to be benefited by their mental and bodily exertions.

